# Fibrous Tumour Mistaken for Aneurism

**Published:** 1844-05-04

**Authors:** 


### FIBROUS TUMOUR MISTAKEN FOR ANEURISM.

A soldier, nineteen years of age, was admitted into the hospital, under M. Engelhardt, with a swelling in the left ham, as large as a goose's egg, partly compact, partly fluctuating, and distinctly pulsating on moderate pressure. This, he said, had shewn itself suddenly while walking, about three weeks previously. Compression was at first tried, and afterwards the femoral artery was tied. The tumour continued to grow, notwithstanding. The patient presented symptoms of diseased lungs, and gradually sunk. On examining the body, the internal organs were found healthy, except the right lung, which was completely converted into a brain-like mass. The tumour in the ham was fifteen inches long. and twenty-four inches in circumference: it was pear shaped, and lobed, and adhered firmly to the periosteum of the femur and tibia. On its surface were five sacculi, holding between five and six ounces of a sero-sanguinolent fluid, and apparently formed by the separation of the two layers, of which a tough fibrous membrane enveloping the whole tumour, was composed. The tumour itself consisted in part of a dry fibrous reticulated tissue, divided by partitions of looser tissue into lobes, and separated into two chief portions by a plate of bone; and in part, of a softer substance, easily broken, rose-coloured, interspersed with fibrous tissue, and containing many cells filled with serous fluid. The femur and tibia were healthy: so also was the joint, except that there was a small mass of soft substance, like that just described, on the inner surface of the synovial membrane. The artery ran over the tumour, and between two of the sacs of fluid on its surface: and through these its pulsations had been so communicated, as to give the sensation of the whole tumour pulsating.

